# Disparity in depressive symptoms between heterosexual and sexual minority men in China: The role of social support

**DOI:** 10.1371/journal.pone.0226178

**Published:** 2020-01-06

**Authors:** Jingchu Hu, Ling Tan, Gang Huang, Wenjun Yu

**Affiliations:** 1 Department of Anxiety Disorders, Shenzhen Kangning Hospital, Shenzhen, Guangdong, China; 2 School of Management, Guangdong University of Technology, Guangzhou, Guangdong, China; 3 Shenzhen Kangning Hospital, Shenzhen, Guangdong, China; 4 Normal College, Shenzhen University, Shenzhen, Guangdong, China; 5 College of Education, JingGangShan University, Ji’An, Jiangxi, China; University of Washington, UNITED STATES

## Abstract

Accumulating evidence suggests that sexual minorities experience elevated levels of depressive symptoms compared to heterosexuals in Western countries. Still, little is known about whether there is any difference in depressive symptoms between sexual minority men and heterosexual men in China. This study investigated the differences in depressive symptoms and social support between 302 Chinese sexual minority men and 249 heterosexual men. The association between depressive symptoms, social support and sexual orientation was also explored. Our results indicated that Chinese sexual minority men have more depressive symptoms and perceived lower social support than heterosexual men. Overall, sexual orientation and social support both predicted depressive symptoms. Different from previous Western studies, in our results, social support fully mediates but does not moderate the relationship between sexual orientation and depressive symptoms in Chinese men. The current findings suggest that social support has a significant impact on depressive symptoms among Chinese sexual minority men, highlighting the unique role of social support in understanding depressive symptoms among Chinese sexual minority men. Providing more social support, as well as promoting accepting and positive environments, may lead to better adjustment in this population in China.

## Introduction

Several studies have demonstrated that sexual minority individuals are at a greater risk for mental health disparities compared with their heterosexual peers [1–6]. Evidence from Western countries suggests that sexual minorities may be preferentially vulnerable to depressive symptoms [1–5], and these symptoms tend to persist throughout their life course [7]. Recent Chinese studies also indicate that Chinese sexual minority individuals are more likely to report psychological problems [8–12]. However, the mental health of Chinese sexual minorities and related influencing factors remain largely unknown. In previous studies, the higher levels of depression among sexual minority individuals has usually been understood by using a minority stress model conceptualized by Meyer, which relates to the unique stressors such as experiences of discrimination, incidents of prejudice, experiences of rejection, internalized homophobia, stigma and concealment [13]. Recent studies have proved that minority stressors (i.e., coming-out stress, sexual stigma) are associated with adverse mental health and higher levels of depression [14–16].

Despite the stress imposed by their sexual minority identity, it may be inappropriate to identify all sexual minority individuals as being at high risk for mental health problems. There remain some protective factors such as social support, which is defined as the perception or experience that one is loved and cared for, esteemed and valued and part of the social network of mutual assistance and obligations [17]. Social support can also provide stress-buffering effects against multiple stressors such as events of physical violence, emotional abuse, and prejudice [18,19]. Adequate social support will offset or moderate the negative impact of minority stress on mental health. For example, social support has been found to serve as a buffer against the negative effects of minority stress and depressive symptoms among sexual minorities [20,21]. However, sexual minority individuals are more likely to suffer low levels of social support [22], which results in impaired mental health. Safren and Heimberg [23] examined the factors related to depression in LGB adolescents and concluded that a lack of social support plays a major role in predicting psychological distress in this population. Vincke and Bolton [24] found that low levels of social support associated with higher levels of depressive symptoms. Thus, social support is particularly essential for the mental health of sexual minority individuals and may plays a “buffering” role in coping with sexual minority stress.

In China, from at least the twentieth century, homosexuality was banned until it was decriminalized in 1997. Although homosexuality was formally removed as a mental disorder in 2001, Chinese same-sex couples still have no legal right to marry or adopt children, in contrast to many countries and states in the West that have legalized same-sex marriage for sexual minority couples. It remains difficult for Chinese people to accept same-sex orientation and behaviours [25]. Recent studies have shown that attitudes toward homosexuality in China remain negative and that this bias continues [26]. Such a negative attitude may create a stressful social environment for Chinese sexual minority people, who face unique stigma from family and society [25,26]. As a result, Chinese LGB individuals may suffer more sexual minority stress and are therefore more likely to suffer from mental health problems than their heterosexual counterparts. Our data from China suggest that Chinese youths who experienced poorer mental health status may be more likely to have a weaker sense of meaning in their life, which promotes greater suicidal ideation [27]. It is important to understand and enhance the mental health of this Chinese minority group.

However, sexual minority studies in China are still limited. No one has explored the differences in depressive symptoms and social support between heterosexual men and sexual minority men in China. Therefore, the aims of this study were to compare sexual orientation differences in depressive symptoms and social support and to explore the possible association between sexual orientation, social support, and depressive symptoms in Chinese sexual minority men and Chinese heterosexual men. Our first hypothesis is that Chinese sexual minority men will report more depressive symptoms and perceive lower levels of social support and, of subtypes of social support (from family, friends, and significant other) than Chinese heterosexual men. In addition, we hypothesize that the level of social support from family will be lower than the social support from friends and from significant other among Chinese sexual minority men; we expect that, these differences will not be found in heterosexual men. Next, given the large body of evidence suggesting that social support is associated with mental health [17–24], we hypothesize that social support will be inversely associated with depressive symptoms in Chinese men. Finally, based on previous research, social support could serve as a moderator and a mediator in the relationship between sexual minority identity and mental health [19–24]. We expect to find that social support moderates and mediates the associations between sexual orientation and depressive symptoms.

## Materials and methods

### Participants

A total of 596 Chinese participants between the ages of 14 and 64 took part in our study. In the data analysis, females (N = 33) were excluded due to the small number of participants; we also excluded participants who were younger than 16 years old (as they may not have yet developed a stable sexual identity/orientation) (N = 12). The final sample consisted of 302 sexual minority men (259 men self-identified as gay, 43 men as bisexual; Mean age = 24.2, SD = 4.6) and 249 men self-identified as heterosexual (Mean age = 24.0, SD = 4.2).

### Procedure

This study was conducted online via a Chinese survey website (www.sojump.com). Sexual minority participants were recruited from websites of sexual minorities (including gay groups on douban.com and feizan.com) and gay social groups on QQ (a popular social application in China). Similarly, heterosexual participants were recruited online from general social websites and forums (a local social network from douban.com, BBS and QQ groups of universities). Participation was voluntary, incentivized by the opportunity to win a small amount of money (¥10) and to learn about their psychological traits. Prior to the survey, each participant was presented with the purpose of this survey and a question of consent: Do you understand the purpose of this study and agree to participate in? The survey will continue if they chose "Yes, I understand the purpose of this study and agree to participate in", The survey will end automatically if they chose "No, I don't agree to participate in this study". Institutional ethics approval for the study was granted by the Normal College, Shenzhen University.

### Measures

#### Sociodemographic variables and sexual orientation

The sociodemographic variables in this study included age, whether the participant was an only child, marital status, education level, occupation, and monthly income. Table 1 shows the sociodemographic characteristics of the participants. Chi-squared analyses and independent t-tests indicated that there were no significant differences between the groups with regard to age, only child, occupation, or monthly income. The only significant difference was found in marital status: more heterosexual men were married compared to sexual minority men. To determine the sexual orientation of the participants, they were asked “What is your sexual orientation?” Participants responded by choosing either “Heterosexual,” “Homosexual” or “Bisexual” from a drop-down list.

**Table 1:** Sociodemographic characteristics of the sample (n = 551).

Demographic variables	Heterosexual men (N = 249)	Sexual minority men (N = 302)	*x2* or *t*
Age (mean ±SD)	24.2±4.6	24.0±4.2	0.89
Whether only child *n* (%)			1.10
Only child	134(53.8)	149(49.3)	
Has siblings	115(46.2)	153(50.7)	
Marital status (opposite sex) *n* (%)			42.59***
Single	205(82.3)	294(97.4)	
Married	43(17.3)	5(1.7)	
Separated/divorced	1(0.4)	2(0.7)	
Widowed/widower	0	1(0.3)	
Education level *n* (%)			3.89
Junior high school or lower	4(1.6)	2(0.7)	
Senior high school	15(6.0)	21(7.0)	
College	214(85.9)	248(82.1)	
Post-graduate or higher	16(6.4)	31(10.3)	
Occupation *n* (%)			0.36
Student	116(46.6)	133(44.0)	
Employee	133(53.4)	169(56.0)	
Income (¥/month) *n* (%)			1.76
<2,000	112(45.0)	120(39.7)	
2,000–6,000	97(39.0)	126(41.7)	
6,000–10,000	25(10.0)	37(12.3)	
>10,000	15(6.0)	19(6.3)	

**** *p* < 0.001

#### Depressive symptoms

A Chinese translated version of the Center for Epidemiological Studies-Depression Scale (CES-D) was used to measure daily mood and other related depressive symptoms. The CES-D consists of 20 items measured on a Likert-type scale, ranging from 0 to 4 (0 = rarely or none of the time, 4 = most or all of the time). Previous studies have found the CES-D to be a valid and reliable measure of depressive symptoms [28]. Studies on the reliability and validity of the CES-D among Chinese [29,30] showed Cronbach's alphas above 0.80, and the CES-D had high correlations with similar questionnaires such as the SDS [31], Beck Depression Inventory, and Depressive Experiences Questionnaire [32]. In this study, the Cronbach's α was 0.78 for the entire sample, 0.75 for Chinese sexual minority men, and 0.82 for Chinese heterosexual men.

#### Social support

We used the Multidimensional Scale of Perceived Social Support (MSPSS) [33] to assess perceived social support. The scale consists of 12 items to which participants respond using a Likert-type scale ranging from 1 to 7 (0 = very strongly disagree, 7 = very strongly agree). The 12 items are divided into three 4-item subscales to assess three sources of social support (family, friends, and significant other). Thus, participants receive three subscale scores and a total scale score, with higher scores indicating higher perceived social support from their social networks. Huang translated the MSPSS into the Chinese version and examined the components of the MSPSS using factor analysis [34]. The Chinese version of this scale has been widely used in China and has demonstrated good internal consistency and reliability, with Cronbach’s alphas of 0.87, 0.85, 0.91, and 0.88 for family, friends, significant other, and total scale, respectively, and with good stability coefficients (0.85, 0.75, 0.72, and 0.85, respectively) as well [35]. In this study, the Cronbach’s α was 0.91 for the entire sample, for both Chinese sexual minority men and heterosexual men.

### Statistical analysis

First, Chi-square analysis was used to test differences in demographic variables (whether an only child or not, education level, marital status, occupation, and personal monthly income) between Chinese sexual minority men and heterosexual men. The next step was to determine whether Chinese sexual minority men report more depressive symptoms and lower levels of social support than their heterosexual peers. Using independent sample t-tests, we compared mean levels of depressive symptoms, total social support and subtypes of social support (from family, friends, and significant other) by sexual orientation. Multiple comparisons (Bonferroni corrected) were performed to compare the different subtypes of social support (from family, friends and significant other) among Chinese sexual minority men and Chinese heterosexual men, respectively. We used dummy-coded variables for sexual orientation, in which the value of 1 indicates sexual minority. Bivariate correlations were also calculated between the sexual orientation dummy variable, depressive symptoms, and social support among all participants, sexual minority men, and heterosexual men. Lastly, potential moderating effects were examined using a stepwise hierarchical regression analysis approach. Potential mediating effects were assessed using the bootstrapping method [36,37]. Demographic variables (age, whether an only child or not, education level, marital status, occupation, and personal monthly income) were included as covariates in the moderation and mediation analyses. Whether an only child or not, education level, marital status, occupation and personal monthly income were operationalized as a series of dummy coded variables. All analyses in this study were performed using IBM SPSS for Windows (Version 18.0).

## Results

### Depressive symptoms, social support between heterosexual and sexual minority men

Means and standard deviations for depressive symptoms, overall social support, social support from family, social support from friends, and social support from significant others are shown in Table 2. Chinese sexual minority men reported more depressive symptoms, *t*(549) = -2.62, *p* = 0.009, Cohen's d = 0.22, lower levels of social support (overall), *t*(549) = 5.58, *p* < 0.001, Cohen's d = 0.50, lower levels of social support from family, *t*(544.96) = 9.46, *p* < 0.001, Cohen's d = 0.80, lower levels of social support from friends, *t*(545.04) = 2.20, *p* = 0.029, Cohen's d = 0.19, and lower levels of social support from significant others, *t*(549) = 1.87, *p* = 0.063, Cohen's d = 0.16, than did Chinese heterosexual men. In particular, the one-way ANOVA showed a significant difference between subtypes of social support among Chinese sexual minority men, *F* (2,903) = 36.89, *p* < 0.001, and among Chinese heterosexual men, *F* (2,744) = 4.66, *p* = 0.01. Multiple comparisons using the Bonferroni post hoc criterion for significance indicated that Chinese sexual minority men's social support from family was lower than their social support from friends (*p* < 0.001) and, from significant others (*p* < 0.001). Further, their social support from significant others was lower than that from friends (*p* = 0.047). However, these differences could not be found in heterosexual men. Chinese heterosexual men's social support from family was higher than that from significant others (*p* = 0.025) and their social support from friends was also higher than that from significant others (*p* = 0.025). No significant differences were found between social support from friends and that from family among Chinese heterosexual men (*p* > 0.05). These results indicate that Chinese sexual minority men felt they were receiving the most support from their friends and the least from their families, while Chinese heterosexual men perceived equivalent social support from family and friends. The latter group also perceived social support from both family and friends as being higher than that received from significant others.

**Table 2:** Means and correlations of outcome, predicators, and covariates.

Variable	Mean	SD	Correlations				
			1	2	3	4	5
**Overall**							
1. Depressive symptoms^c^	38.55	10.76	-				
2. Social support^c^	58.56	13.22	-0.41***	-			
3. Social support from family^c^	18.57	6.02	-0.35***	0.80***	-		
4. Social support from friends^b^	20.54	4.74	-0.36***	0.87***	0.48***	-	
5. Social support from significant others^a^	19.45	4.92	-0.32***	0.87***	0.47***	0.79***	-
6. Sexual orientation^d^	-	-	0.11**	-0.23***	-0.37***	-0.092*	-0.08
**CSMM**							
1. Depressive symptoms	39.63	10.91	-				
2. Social support	55.78	13.35	-0.40***	-			
3. Social support from family	16.54	5.86	-0.34***	0.78***	-		
4. Social support from friends	20.15	4.95	-0.33***	0.87***	0.43***	-	
5. Social support from significant others	19.10	5.07	-0.35***	0.89***	0.48***	0.80***	-
6. Sexual orientation	1	-	-	-	-	-	-
**CHM**							
1. Depressive symptoms	37.24	10.45	-				
2. Social support	61.93	12.27	-0.39***				
3. Social support from family	21.02	5.26	-0.33***	0.81***	-		
4. Social support from friends	21.02	4.44	-0.40***	0.89***	0.56***	-	
5. Social support from significant others	19.88	4.70	-0.28***	0.86***	0.47***	0.77***	-
6. Sexual orientation	0	-	-	-	-	-	-

Note: CSMM = Chinese sexual minority men, CHM = Chinese heterosexual men

^a^ Results of independent sample t-test between CSMM and CHM, no significant difference

^b^ Results of independent sample t-test between CSMM and CHM, significant difference at the 0.05 level

^c^ Results of independent sample t-test between CSMM and CHM, significant difference at the 0.01 level

^d^ Binary variables are coded as 0 and 1, where the value of 1 indicates sexual minority

*p < 0.05

**p < 0.01

***p < 0.001

Table 2 also shows the correlations between depressive symptoms, social support, and sexual orientation dummy variable. For all participants, depressive symptoms were significantly negatively correlated with overall social support, *r* = -0.41, *p* < 0.001, social support from family, *r* = -0.35, *p* < 0.001, social support from friends, *r* = -0.36, *p* < 0.001, social support from significant others, *r* = -0.32, *p* < 0.001, and sexual orientation dummy variable, *r* = 0.11, *p* = 0.009. For Chinese sexual minority men, depressive symptoms were significantly negatively correlated with overall social support, *r* = -0.40, *p* < 0.001, social support from family, *r* = -0.34, *p* < 0.001, social support from friends, *r* = -0.33, *p* < 0.001, and social support from significant others, *r* = -0.35, *p* < 0.001. Similarly, significant negative correlations were found in Chinese heterosexual men. Depressive symptoms negatively correlated with overall social support, *r* = -0.39, *p* < 0.001, social support from family, *r* = -0.33, *p* < 0.001, social support from friends, *r* = -0.40, *p* < 0.001, and social support from significant others, *r* = -0.28, *p* < 0.001. These results indicate that higher scores for depressive symptoms were significantly related to lower levels of social support (overall, from family, from friends, and from significant others) among all participants. Minority sexual orientation was significantly related to higher scores for depressive symptoms and lower levels of social support.

### Social support as a moderator

In order to test the potential moderating effects of social support on the relationship between sexual orientation and depressive symptoms in sexual minority men and heterosexual men, hierarchical regression procedures were performed. The demographic variables (age, whether an only child or not, education level, marital status, occupation, and personal monthly income) were controlled for in the moderation model and, were entered into the first block of the regression analysis. As shown in Table 3, the interaction of sexual orientation dummy variable and social support entered in Block 4 explained 17.9% of the variance in depressive symptoms, which is lower than Block 3 (18.1%). In addition, the β of interaction was not significant in Block 4 (p = 0.666). Therefore, this model did not account for significant variance in depressive symptoms, and moderation effects were not significant.

**Table 3:** Hierarchical regression analysis on depressive symptoms among Chinese men.

Predictor variables	B	SE B	*β*
**Block1-Demographics**			
Age	0.16	0.13	0.07
Whether only child			
*Has siblings*	1.13	0.94	0.05
Education level			
*Senior high school*	0.64	4.74	0.02
*College*	-3.07	4.41	-0.12
*Post-graduate or higher*	-3.24	4.70	-0.08
Income(¥/month)			
*2*,*000–6*,*000*	-1.60	1.35	-0.07
*6*,*000–10*,*000*	-0.44	1.93	-0.01
*>10*,*000*	-5.45	2.28	-0.12*
Marital status			
*Married*	-4.44	1.82	-0.12**
*Separated/divorced*	3.15	6.30	0.02
*Widowed/widower*	-16.15	10.90	-0.06
Occupation			
*Employee*	0.86	1.45	0.04
*F*(12,538) = 1.94*; Adjusted *R*^2^ = 0.020			
**Block 2-Sexual orientation**	1.93	0.96	0.09*
*F*(13,537) = 2.11*; Adjusted *R*^2^ = 0.026			
**Block 3-Social support**	-0.33	0.03	-0.41***
*F*(14,536) = 9.66***; Adjusted *R*^2^ = 0.181			
**Block 4-Sexual orientation× Social support**	-0.03	0.07	-0.08
*F*(15,535) = 9.02***; Adjusted *R*^2^ = 0.179			

** *p* < 0.01

*** *p* < 0.001.

### Social support as a mediator

**Fig 1:**
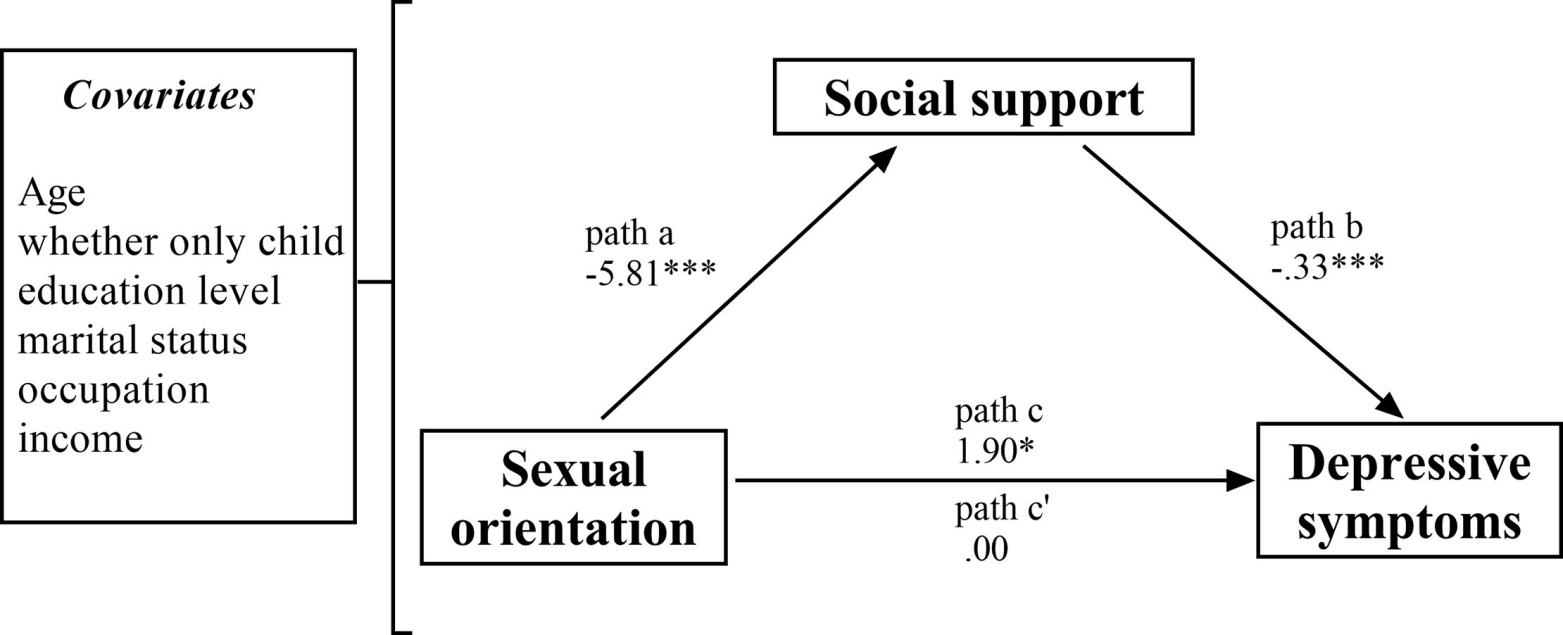
Mediation analysis of sexual orientation and social support on depression symptoms. **p < 0.01; ***p < 0.001.

In our next analysis, we tested whether social support is a mediator in the relationship between sexual orientation and depressive symptoms. The bootstrapping procedure was employed to assess the mediation effect with demographic variables (age, whether an only child or not, education level, marital status, occupation, and personal monthly income) included in the model as covariates. As described by Preacher and Hayes [37], we tested the direct and indirect effects with 1,000 bootstrapping samples. The model with path coefficients is presented in Fig 1. Bootstrapping estimates revealed that social support had a significant indirect effect on the relationship between depressive symptoms and sexual orientation. More specifically, the total (path c) and direct effects (path c’) of sexual orientation dummy variable on depressive symptoms were 1.897, *p* = 0.049 and 0.003, *p* = 0.998, respectively. The difference between the total and direct effect was different from zero based on the point estimate of 1.89 (SE = 0.45) and a 95% Bias Corrected and Accelerated (BCA) bootstrap CI of 1.02 to 2.79. These results showed that social support fully mediated the relationship between sexual orientation and depressive symptoms. Given the different levels found between subtypes of social support among Chinese sexual minority men and among Chinese heterosexual men, each subtype of social support (social support from family, friends, and significant other) was tested as a potential mediator of the relationship between sexual orientation and depressive symptoms while controlling for the demographic variables. Overall, social support from friends and social support from significant others did not significantly mediate the relationship between sexual orientation and depressive symptoms (the 95% confidence interval includes zero). Only social support from family still had a significant indirect effect on the relationship between depressive symptoms and sexual orientation (point estimate = 2.69, BCA bootstrap CI = 1.75 to 3.86). The total (path c) and direct effects (path c’) of the sexual orientation dummy variable on depressive symptoms were 1.897, *p* = 0.049 and -0.791, *p* = 0.412, respectively. These follow-up analyses suggested that the full mediation effect of total social support on the relationship between sexual orientation and depressive symptoms was mainly driven by social support from family.

## Discussion

This study is the first to specifically compare the level of depressive symptoms and social support between Chinese sexual minority men and Chinese heterosexual men and to examine the role of social support and sexual orientation in depressive symptoms among Chinese men. Consistent with our first hypothesis, we found that Chinese sexual minority men reported more depressive symptoms than their heterosexual counterparts. These results were consistent with previous findings conducted in Western populations, in that sexual minority individuals showed more psychological distress than heterosexual individuals [38–40], and provide new evidence that sexual orientation differences in mental health tend to be cross-cultural.

It is worth noting that Chinese sexual minority men received lower overall social support than Chinese heterosexual men from family, friends, and significant others. Moreover, Chinese sexual minority men received the least social support from their families compared to social support received from friends and significant others. In contrast, Chinese heterosexual men received more social support from their families than from significant others. They also received similar levels of social support from their families and friends. Our results are partly consistent with lower social support levels reported by sexual minority individuals in previous research [41,42]. Although we found that Chinese sexual minority men received lower levels of social support from friends than heterosexual men, they nevertheless reported that they received the highest level of social support from friends, which is consistent with previous studies that sexual minorities' friends provide the highest level of social support [43,44]. These results add new evidence of the importance of friends in providing social support for sexual minority men.

Our current study found substantial support for the hypothesis that social support is associated with depressive symptoms, and sexual orientation among Chinese men. Minority sexual orientation was associated with low levels of social support (overall, from family, friends, and significant others) and high levels of depressive symptoms. We confirmed the well-established relationship between psychological distress and a non-heterosexual orientation [45,46]. Different from our hypothesis and previous Western studies, we found that social support fully mediated (but not moderated or partly mediated) the relationship between sexual orientation and depressive symptoms among Chinese sexual minority men and Chinese heterosexual men. When controlling for social support, the link between depressive symptoms and sexual orientation was no longer significant. In particular, social support from family (one of the social support subtypes) is primarily responsible for this full mediation effect. These results demonstrate the powerful influence of social support, particularly social support from family, on the negative health-related outcomes reported by Chinese sexual minority men. Several cultural factors may help to explain the crucial role of social support in depressive symptoms among Chinese sexual minority men. First, procreation and continuation of one's paternal family lineage are the undeniable duties of every Chinese man in traditional Chinese Confucian culture [47]. However, Chinese sexual minority men may fail to fulfil these familial and social expectations. As a consequence, their social environment reacts negatively and attempts to force them to conform by threatening to withdraw social support. In some cases, this provokes considerable stress and stigma, possibly leading to depressive symptoms [14]. Second, people in a collectivistic culture such as that existing in China are expected to form a connected entity, with individuals and families bound to one another in a culture that considers group or family goals as primary and personal needs/beliefs as secondary. Chinese sexual minority men may conceal their sexuality and request social support more cautiously, not wanting to bring their personal problems into the open [48].

### Strengths and limitations

Overall, our findings provide important confirmation and extension of previous findings. The comparison between Chinese heterosexual men and sexual minority men in this study was the first to suggest a number of consistencies with the differences that are well-documented in Western samples. Our study is also the first to provide an integrated exploration of the relationship between sexual orientation and psychological well-being in China, highlighting the unique role played by social support in the mental health of the Chinese population.

However, several limitations of the present study must be noted. First, the sole use of self-report measures and a nonrandom sample of Chinese sexual minority participants recruited via the Internet represent methodological limitations. Only those who utilize the Internet or use gay social networking apps and websites were involved in the survey, which can lead to a certain bias. In addition, most participants recruited were college students or graduates who had just started working (considering their incomes); thus, our results might not be generalizable to people who are from rural areas or lack a college degree. Additionally, we did not include questions regarding the participants’ hometown or residential status in the survey, which restricts the generalizability of the results. It is also worth noting that most sexual minority participants in this study were gay men. Caution should be used in extending the present findings to bisexual men because the stress on and social support for bisexual men may differ. Lastly, the cross-sectional design of the study limits the causal implications that can be drawn from the results.

## Conclusions

In this study, we found that Chinese sexual minority men reported more depressive symptoms and perceived lower social support than heterosexual men. Sexual orientation and social support both predicted depressive symptoms, while social support fully mediated the relationship between sexual orientation and depressive symptoms. The current study supports a contextual model of psychological well-being among Chinese sexual minority men. Lack of social support appears to be the prominent factor contributing to emotional difficulties, rather than sexual orientation. By stressing the importance of social support, the present findings may contribute significantly to decreasing the level of depressive symptoms among Chinese sexual minority men. Further studies should continue to examine the factors that explain mental health problems in Chinese sexual minority population. Moreover, intervention such as psychosocial skills training, which proved to be an effective means of reducing depressive symptoms and increasing social support in samples of Chinese [49, 50], should be provided to improve mental health of Chinese sexual minority men. The promotion of more positive and accepting social environments, as well as an appreciation of the importance of the family relationships and friendships of Chinese sexual minority men, may improve the mental health of this population.

## Supporting information

S1 Database SPSS database of responses to survey questions. (SAV)
